# Leaping Forwards, Bouncing Forwards, or Just Bouncing Back: Resilience in Environmental Public Agencies Through after the Austerity Decade

**DOI:** 10.1007/s00267-022-01701-z

**Published:** 2022-08-25

**Authors:** Nick Kirsop-Taylor

**Affiliations:** University of Exeter, Exeter, Devon UK

**Keywords:** Organisation, Austerity, Natural England

## Abstract

The resilience of public environmental agencies is an important but broadly under-researched discourse. This paper addresses this lacuna by drawing on a three-part typology of resilience from organizational studies and applying it to the English natural environment agency, Natural England, following a decade of public sector agency de-funding under the aegis of austerity. The research question was explored qualitatively through eleven semi-structured interviews with the senior management team of Natural England during the summer of 2020. The findings suggest that public agency multi-functionality equate to heterogenous resilience across agency functions; that generally agency resilience (as a function of capacities) is poor with consequences upon good governance; and that they are broadly poorly positioned for the aftermath of Covid-19. The findings speak directly to the regulatory and organizational literatures with public administration by evidencing the complex realities of understanding resiliencies in large multi-functional public environmental agencies.

## Introduction

There has been a marked scholarly interest in recent years into the subject of *environmental resilience* (Fleming and Ledogar 2008; Brown 2013; Whyte 2014). Some of these discourses and scholarship have focused on questions about ecological resilience (Mori et al. 2013; Chambers et al. 2019), resilience in environmental management (Benson and Garmesani 2010; Marchese et al. 2018), resilience in indigenous peoples (Ford et al. 2020) and as a dynamic component in sustainability transitions (Wilson 2012). Concomitantly, there has been a marked upturn (or return) to the resilience discourse in organizational studies (Lengnick-Hall et al. 2011; Limnios et al. 2014; Duchek 2020), a movement exacerbated by the Covid-19 pandemic (see the multiple contributions in Ramanathan et al. 2021). However, there are very few contributions seeking to bring the “environment” and “organization” streams of scholarly activity together; in so doing offer accounts of why scholars and practitioners should be interested in the resilience of environment-facing organizations. Certainly, there is small literature (see: Den Uyl and Russel 2018; Kirsop-Taylor and Russell 2021) noting the disproportionate impacts upon environmental regulatory public agencies from resilience-reducing perturbations, such as significant and/or sustained defunding, complexity challenges, institutional and regulatory fragmentation. And whilst other scholarship takes account of the effectiveness of contemporary environmental public agencies (Heald and Steel 2018; Den Uyl and Russel 2018; Kirsop-Taylor et al. 2020) questions relating to the resilience of public organizations remain broadly unaddressed. This is an important discussion and problematic lacuna because, as other scholarship has shown (Koontz et al. 2004; Kooiman et al. 2005; Kirsop-Taylor et al. 2020), environmental public agencies are key actors in contemporary environmental management and governance (a detailed account is given by Koontz et al. 2004). And the absence or disruption of public environmental agencies participating in environmental management due to their poor resiliencies is likely to cause significant disruptions to the dominant contemporary modality of partnership-based environmental management. This paper speaks to this lacuna through a theoretically informed empirical case study of organizational resilience within a national-scale environmental management-facing public agency in the near aftermath of a prolonged period of defunding and institutional-regulatory change. This paper offers a consequential contribution and discussion for environmental management scholars and practitioners concerned with issues of public agency capacities, competencies, and general resilience as they relate to the good governance and management of natural environments. It does this through posing a key question:

How resilient has a public environmental agency been to the austerity-decade as a major perturbation?

To address this question I use Stephanie Duchek’s (2020) three-part capacity framework of organizational resilience in the context of environmental public regulatory agencies. Duchek’s framework summarizes the long and rich organizational studies literatures on organizational resilience under perturbation. These literatures (see: Meyer 1982 and others) are summarized in terms of offensive, defensive, and anticipatory resilience. In the context of this paper—these three forms of resilience are characterized as a public environmental agency’s capacity-orientated ability(ies) to “leap forwards, bounce forwards, or bounce back”, and in so doing addresses the principal research question.

The paper progresses as follows, section two outlines the theoretical and governance context of the paper in environmental agency governance in the United Kingdom (UK) after a decade of austerity defunding; and an exploration of Duchek’s framework (2020). Section three outlines the research design and methodology used in the empirical component of the paper, and section four presents an overview of the qualitative findings. Section five analyzes the findings in response to theory and research question. It concludes in section six where the future of agency resilience in the post-covid period is explored based on the findings of the study.

## Theoretic and Governance Contexts

The Covid-19 pandemic has brought into focus the resilience of public environmental agencies operating under extremely challenging operational environments (Khan et al. 2020, Kirsop-Taylor and Russell 2021). Many of these agencies were children of the agency governance agenda (e.g., Verhoest 2013) that sought to ease the burdens on central government(s) through sharing greater expert-led governance across a wider spectrum of actors (Elston 2012). This agency governance model normatively brings order, uniformity, impartiality, and expertise to business of governance (ibid.). Whilst there has been a global proliferation in agency-government since the 1990s (Levi-Faur 2010), and in agency forms (Jordana et al. 2018), many scholars highlight challenges to this normativity through organizational fragmentation (Den Uyl and Russel 2018), under-funding (Heald and Steel 2018) and increasing politicization (Ennser-Jedenastik 2016). Furthermore, these agencies increasingly operate under complex trans-disciplinary accountability regimes (Kirsop-Taylor and Hejnowicz 2020) that defy the logics of siloed good agency governance (e.g., the siloed Weberian ideal type—see: Russel and Benson 2014). Within these increasingly constrained, complex, and problematic governance environments agencies nevertheless have core delegated functions and accountabilities to meet (Thatcher 2004, Bach 2012), principally orientated around diverse sets of organizational functions such as regulatory functions, advisory functions, governance functions and functions in the policy process (see: Pedersen 1978, Thatcher 2004).

Building on the work of Meyer (1982) and others (e.g., Limnios et al. 2014) Stephanie Duchek (2020) has recently coalesced the organizational literatures to argue that resilience is contingent upon organizational capacities. Others have argued that resilience is not so much an outcome(s) as a continuous anticipatory process (Weick et al. 1999). And recent scholarship has been offering accounts of resilience in the public organizational sphere from many perspectives (Van de Walle 2014, Capano and Woo 2016, Plimmer et al. 2021). Such as in the relationships between agency resilience and leadership (Stewart and O’Donnell 2007, Franken et al. 2020, Plimmer et al. 2021) and preparedness (Khan et al. 2020; Samsuddin et al., 2018). However, others have noted the unexpectedness of the Covid-19 pandemic that defied forward-facing resilience (Bryce et al. 2020).

Pandemic preparedness is an important duty of government (National Risk Register, 2020: 45–63). Implicit within this is the need for assessing the robustness of the institutions of governance to risk, and the Covid-19 pandemic has re-ignited discourse (ibid: 50–59) about the resilience of public organizations (Dunlop et al. 2020, Plimmer et al. 2021, Ranganathan et al. 2021). And organizational studies have a well-developed and sophisticated organizational resilience literature that we can draw upon and borrow to enhance understandings about contemporary agency governance under perturbation (Meyer 1982, Wildavsky 1991, Limnios et al. 2014, Duchek 2020). Resilience is a long-standing theme in organizational studies, and Duchek (2020), summarizing these literatures notes that:

“To survive in uncertain environments and to foster future success, organizations must be able to handle manifestations of the unexpected”.

**Fig. 1:**
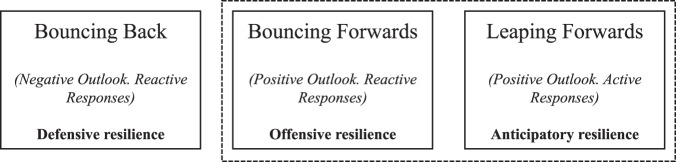
Capacity-orientated organizational resilience framework. Created by author, derived from Duchek (2020). Nature of different resilience type given in parenthesis

This literature has argued that resilience is a key element in contemporary and future organizational success strategies. Resilience is, therefore, in part, about risk and uncertainty management (Somers 2009); but also, the lived experiences of the individuals who make up the organization (e.g., Plimmer et al. 2021). Some in this literature conceptualize resilience as a multi-dimensional assemblage (Tengblad and Oudhuis 2018) and others as a function of organizational capacities for managing and mitigating change (Duchek 2020). However, staring with the work of Meyer (1982) leading to Duchek (2020) there is a clear line of theorization within this literature orientating around a three-part conceptual typology and analytical framework. These are summarized in Fig. 1 (with authors additions), explored in greater below, and utilized analytically in the empirical sections of this paper in addressing tensions expressed in the research question.

Each form of agency/organizational resilience conceptualized in Fig. 1 can be characterized operationally by an organization’s abilities–capacities for action in response to a perturbation; they may aspire towards “bouncing back”, “bouncing forwards”, or “leaping forwards”. Each of these three states can be further defined through their actor’s agency (e.g., will to-for action) in terms of proactive or reactive responsiveness; and their framing of perturbation as either a problematic event to be eventually overcome (negative outlook) or an opportunity for growth and innovation-led change (positive outlook).

*Defensive resilience* (from Limnios et al. 2014) is arguably one of the first conceptualized forms of resilience in organizational scholarship (see: Horne and Orr 1998, Boin and Eten 2013). It is orientated around resistance to change and perturbations, and the organizational ability to quickly and effectively recover after perturbations. Horne and Orr (1998: 31) describe this as a “*fundamental quality (…) to respond productively to significant change that disrupts the expected pattern of event without engaging in an extended period of regressive behavior*”. Robert (2010) conceptualizes defensive resilience as “*a firm’s capacity to maintain or restore an acceptable level of functioning despite perturbations or failures*”. As Lengnick-Hall et al. (2011) note, resilience under this conception is about “*coping strategies*” and the ability to “*quickly recover*”, or to “bounce back” to pre-perturbation levels and forms of functionality.

*Offensive resilience* relates to notions about organizations abilities for learning and adapting through perturbation. What Lengnick-Hall et al. (2011) define offensive resilience as “*a firm’s ability to effectively absorb, develop situation-specific responses to, and ultimately engage in transformative activities to capitalize on disruptive surprises that potentially threaten organization survival*”. Within offensive resilience scholarship (Limnios et al. 2014) organizations turn perturbation into an opportunity to adapt, integrate and reconfigure (Duchek 2020). Perturbation becomes re-framed as opportunities for creative destruction, and the chance to re-frame it from a negative impactor on extant organizational workings into an opportunity for critical and positive reflection and re-appraisal—or as opportunities to “bounce forward” from perturbation.

A third conceptualization is based around the notion of *anticipatory resilience* (Rerup 2009, McManus et al. 2008, Somers 2009). Under this conception, resilience is less about damage mitigation, or recovering better than before, so much as damage avoidance through foresight, such as Ortiz-de-Mandojana and Bansal (2016:9) who conceptualize anticipatory resilience as the “*incremental capacity of an organization to anticipate and adjust to the environment*”. Whilst some, such as Wildavsky (1991) argued that resilience and anticipation were separate categories and should be conflated; others, such as Kendra and Wachtendorf (2003), argue that anticipation and resilience are not anti-ethical, and that in fact organizations achieve resilience through preparation for perturbations (a point supported by Duchek 2020). And Somers (2009) has taken this further by arguing that anticipatory resilience can be achieved through foresighted risk awareness.

## Environmental Regulatory Governance in the UK under Austerity

The austerity decade (2010–2020) was a substantive perturbation impacting upon UK environmental agencies (Kirsop-Taylor et al. 2020, Office for Environmental Protection 2022). This paper considers the austerity decade as the key lens through which we start considering questions of resilience in environmental public agencies. The global financial crisis in 2008–2009 was one of the most significant shocks to the global economic system, in the last century (Stiglitz 2010). The government of the UK initially responded to this through a program of market stabilization and recapitalization (Dyson 2018); but later a meta-policy program of fiscal austerity (Kirsop-Taylor et al. 2020). This included trimming state expenditures to reduce government spending and ostensibly reduce the public budget deficit (Heald and Steel 2018). This austerity program has been critiqued by Keynesian’s who argue that it was economically wrong-headed (Wren-Lewis 2016); social mobility scholars argued that it simply drove up and exacerbated wealth inequality by stripping away essential public services (Brown 2013); and political scientists (see: Cooper and Whyte 2017, Lobao et al. 2018, Gray and Barford 2018) that it represented an ideology-driven attack on the welfare state itself. Whilst some argue that austerity programs are broadly and historically successful in driving down bloated government expenditure (Konzelmann 2014), in the case of the 2010–2020 austerity program in the UK all budgetary deficit reductions were ameliorated by pandemic public spending. Indeed, other scholars augur a second round of public austerity is on the horizon in response to public pandemic spending (Portes 2020, Warner et al. 2020) with profound consequences for how the government will deliver major environmental change agenda (Office for Environmental Protection 2022).

Environmental governance in the United Kingdom (UK) is a partially devolved concern. This means that whilst the UK retains some powers and duties, many others are addressed at sub-national governance scales in England, Wales, Scotland, and Northern Ireland. Over-arching regulatory governance in the UK is overseen by a ministerial-executive department the Department for Environment, Food and Rural Affairs (Defra) supported by other national-scale agencies such as the Environment Agency managing water and hydrological risks, or the Office for Environmental Protection acting as the primary environmental legal watchdog holding government and agencies to account. The devolved aspects of environmental regulatory governance and enforcement are overseen by a community of arms-length agencies with discrete and clearly codified formal statutory duties and at times overlapping informal interests, for example by Natural Resources Wales in Wales (Kirsop-Taylor and Hejnowicz 2020) and Scottish Natural Heritage in Scotland. The institutional architecture of environmental governance that the devolved nations operate under is a complex intersection from UK, European Union (EU), and devolved sources (Kirsop-Taylor et al. 2020). This means regulatory competencies and duties are multi-source and dynamic, especially in light of the UK’s recent departure from the EU. Indeed, there is evidence that post-Brexit EU-UK environmental regulatory and policy divergence has been initially limited (Baldock and Nicholson 2022) but that it might increase in the years to come, especially if the UK seeks to repudiate key regulatory pillars such as Habitats Directive. Normatively the national ecosystem of environmental public agencies are apolitical, expert-driven regulators; though as described by Finke (2020) the inevitable regulatory overlaps and contestations create an environment ripe for turf wars in which agencies compete for scare funding, political engagement, and over contested ideas and agenda.

## Research Design and Methodology

Early austerity-induced budgetary reductions (2010–2017) fell particularly hard upon Defra who, as one the smallest ministerial department in the UK Government enjoyed lesser authority in securing their budget. Conversely, later in the austerity period (2017–2020) Defra saw a relative rise in budget to account for the repatriation of environmental regulatory functions post-Brexit. During this period Defra was also led by Ministers who appeared keen(er) to adopt leading roles in budget reduction trajectories (Kirsop-Taylor et al. 2020).

## Case Study: Natural England

**Fig. 2:**
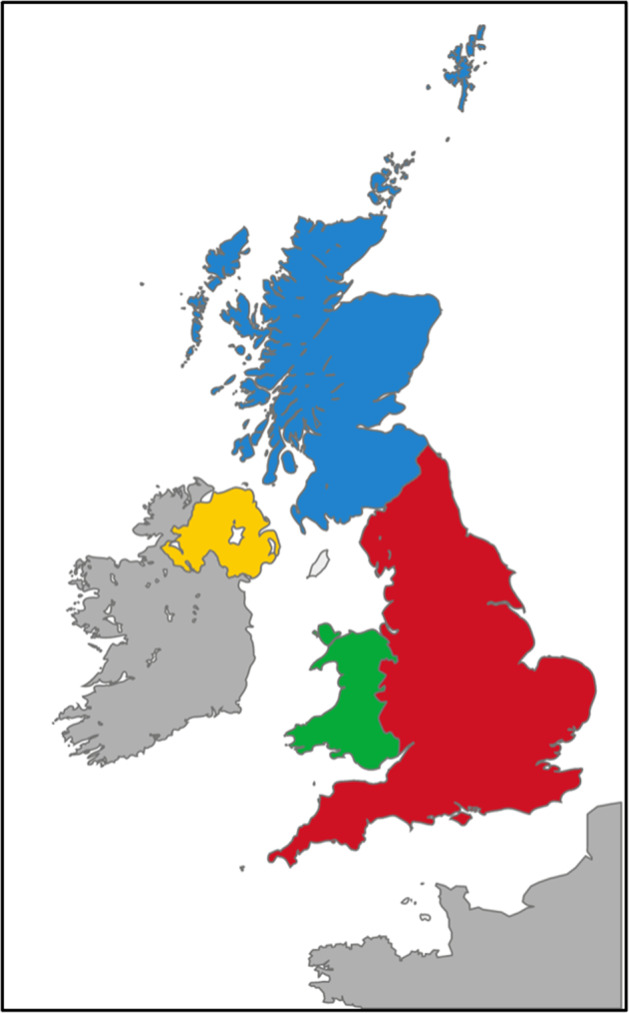
Map of the countries of the United Kingdom. Reproduced from Dank 2021. Red-England, Blue-Scotland, Green-Wales, Yellow-Northern Ireland

Natural England was selected as the case study agency through which the central research question was qualitatively explored. As noted by Koontz et al. (2004), Kooiman (2005) and Kirsop-Taylor et al. (2020) public environmental agencies are key institutional actors in contemporary partnership-based environmental management and governance, and so understanding how Natural England’s resilience has been impacted by the austerity decade could contribute wider understandings about how management is impacted by perturbations to agency resilience. Natural England are a non-departmental public agency “*established by an Act of Parliament in 2006 our purpose is to help conserve, enhance and manage the natural environment for the benefit of present and future generations, thereby contributing to sustainable development*” (UK Gov 2020). Natural England is overseen by the Secretary of State for Environment, Food and Rural Affairs (at time of writing: Rt. Hon. George Eustace MP), and Natural England is sponsored by Defra as its parent department. They are responsible (and accountable) for protecting and enhancing England’s (red section in Fig. 2) natural environment, inclusive of land, water, biodiversity, geology, and soils. The agency employs around 2000 members of staff spread across twelve regional area teams around England and are headquartered in the city of York. Natural England’s work is structured around four strategic outcomes 1) a healthy natural environment, 2) enjoyment of the natural environment, 3) sustainable use of the natural environment and 4) a secure environmental future. Natural England has powers over, and statutory duties across, a broad range of functions. It has the primary duty for the designation and management of England’s Areas of Outstanding Natural Beauty covering 23,301 square km across 46 sites. This involves the active management of the sites. Natural England produces and enforces guidelines under the EU Landscape Convention. Additionally, it prepares and manages heritage coasts and properties. It has duties towards designating and managing Sites of Special Scientific Interest (SSSI), the administration of numerous grant schemes (e.g., the countryside stewardship scheme), powers to define ancient woodlands, managing (some) national nature reserves, awarding grants and many others besides. It acts as a statutory and non-statutory consultee to the Secretary of State for Defra. Perhaps most importantly it has duties under National Parks and Access to Countryside Act (1949) to designate new National Parks, and under the Wildlife and Countryside Act (1984) to change extant National Park boundaries. Natural England is a multi-faceted and essential actor in English national environmental management (as Koontz et al. 2004 envisage) and governance (as argued in Kirsop-Taylor et al. 2020).

The UKs austerity decade was characterized by major financial and regulatory perturbations for Natural England. It precipitated a 57.9% decrease against 2010 reductions in central government Grant in Aid (GiA) for Natural England, which, when adjusted for inflation (taken as a static 2.1%, based on an average Retail Price Index through austerity), evidenced a real terms reduction in GiA of 67.8% compared to 2010. With their GiA being their most substantive revenue source (consistently above 95% of funding). The significant reductions in GiA were delivered without any reductions in statutory duties, meaning similar or rising regulatory enforcement burdens under static or falling budgetary conditions. Budgetary cuts were in the main strategically planned through the Spending Reviews in 2010 and 2015.

**Fig. 3:**
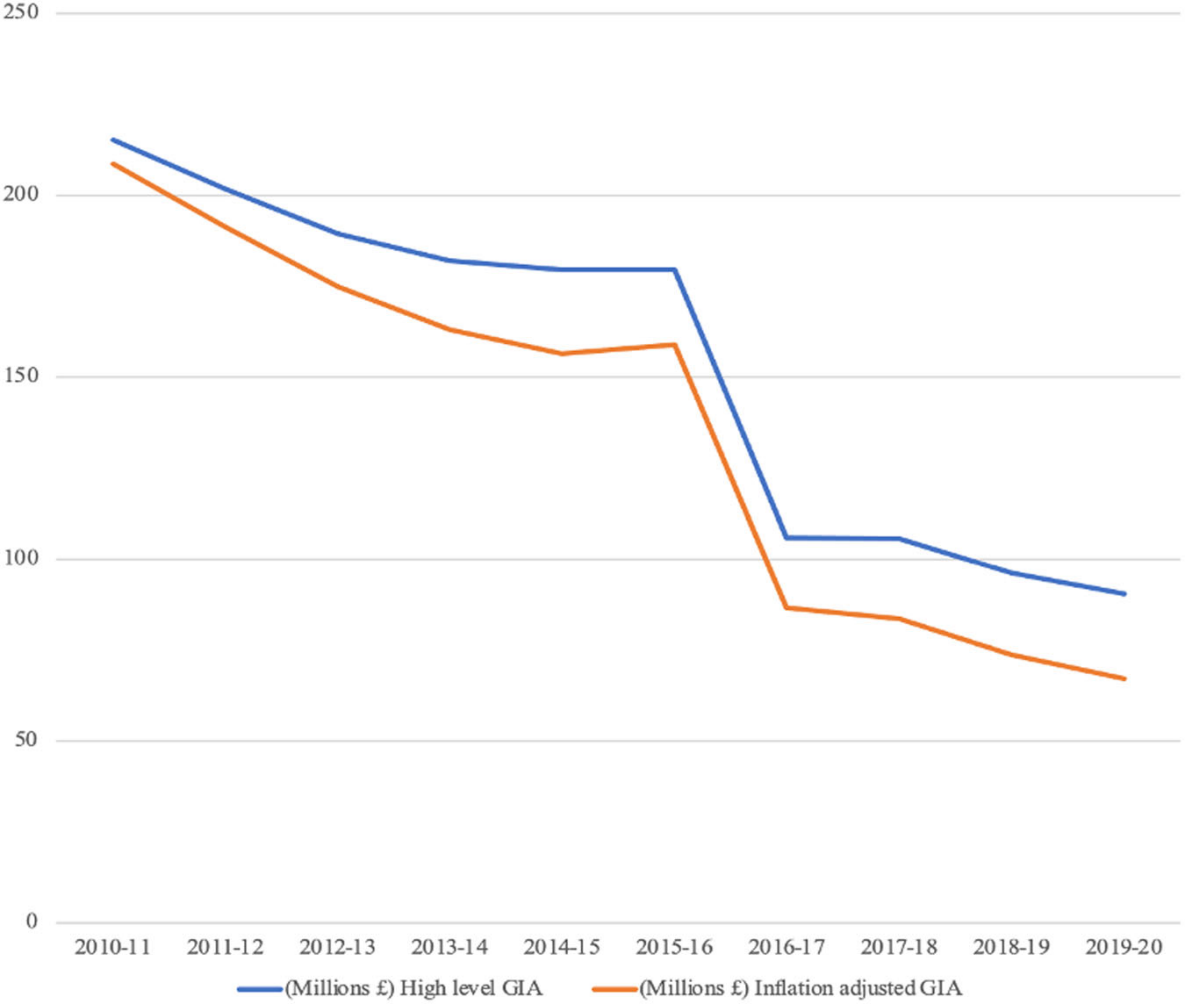
Natural England GiA under the austerity decade (2010–2020). Created by author based on Natural England annual financial reports

The reductions in centrally disbursed GiA seen in Fig. 3 raised serious questions about the capacities of Natural England to continue to deliver their range of statutory and non-regulatory functions. There has been much written and said in recent years about the capacities of agencies to continue to deliver regulatory good governance under the continuing shadow of austerity de-funding (Fuller 2017, Heald and Steel 2018, Kirsop-Taylor et al. 2020). Some scholarship has been devoted to theorizing the structural implications of austerity on regulatory agencies (see: Heald and Steel 2018). And some argue that the diminishment of agencies presages a form of state retreat (Strange, 1996) from governance and regulatory duties (Kirsop-Taylor et al. 2020). Others take a longer view and argue that the austerity decade was merely the latest chapter in the long term “hollowing out of the state” (Rhodes 1994, Skelcher 2000) stretching back to the birth of neoliberalism in 1979 (Wren-Lewis 2022) which can be characterized through its excessive over-reliance on governance, partnerships, and self-regulation leading to reduced centralized/state capacities and competences for governing.

## Methodology

The central research question is explored empirically through the case study of Natural England. Similar research within large multi-functional public agencies has highlighted the value of the elite interview method for understanding complex organizational change situations (Odendahl and Shaw 2001; Kirsop-Taylor and Hejnowicz 2020, Kirsop-Taylor and Russell 2021). Fewer focused interviews with senior managers chosen for their detailed knowledge of a subject are likely to yield richer data than other sampling approaches (ibid.). Kirsop-Taylor and Hejnowicz (2020) have highlighted the utility of elite research designs for capturing hidden and deeply contextual knowledges in such agencies. Elite interviewing brings drawbacks in terms of accessibility, positionality, and small *n* sample sizes (Odendahl and Shaw 2001). These can be offset by the benefits of gaining first-hand detailed and nuanced accounts.

Participants from Natural England enjoyed a high level of anonymity in interviews due to the sensitivity of the questions and subject matter. All participants are senior members of Natural England, with membership of the senior board, no other identifying information about them is disclosed in this paper. The initial contact was established through an existing organizational gatekeeper, which led to opportunity and snowball sampling. The interview sample comprised members of the executive board (*n* = 9) and Directors (*n* = 2). The limited population of organizational elites to sample from were, in all probability, the only interviewees who could offer such detailed insights into the research question under investigation, which justified the small eventual sample size.

The semi-structured interview method facilitated both addressing the central research question whilst giving flexibility for elite-led discourse. Empirical data was collected between May and July 2020 through a combination of Skype-based and Microsoft Teams-based interviews and telephone calls. Interviews were conducted at the height of the Covid-19 pandemic in the UK, and as such were all conducted remotely with no opportunity for face-to-face interview. The remote interviews were recorded using the *italk* application and produced over ten hours of data for transcription and analysis. After digital transcription, the data were analyzed in *NVivo 11* against a partially pre-set, but emergent and iterative node framework based on parent nodes.

## Findings

The interview findings are orientated around 1) reflections on the austerity period and 2) agency multi-functional resiliencies in response to austerity.

### Natural England under Austerity

Interviewees noted the effects of the austerity decade on Natural England in three key forms. First, it had a deleterious effect on their physical abilities to meet some of their statutory regulatory functions. Second, it affected their human capital by forcing staffing reductions and recruitment freezes, reducing their ability to recruit new expert staff to meet new challenge areas, and by reducing the agency's spend on staff training and development programs and promotion trajectories. Thirdly, it damaged the agency’s culture for environmental regulatory enforcement.

Eleven interviewees commented on the implications of reducing GiA through austerity. These cuts had come in waves and had been facilitated by Defra Ministers and a former senior leader within Natural England who, four participants noted, had acted with more alacrity than was needed in forwarding the agency for budget cuts, or as P3 noted “*he showed a little too much zeal in slicing*”. Five interviewees suggested this precipitated a dawning awareness of their inability to physically meet the demands required of certain regulatory functions; evidenced in their gradual retreat from environmental governance decision-making forum and abilities to conduct site visits for governance and/or regulatory purposes. They were increasingly not present in governance and management activities and had retreated back towards less consensual interpretations of their statutory duties. For example, P7 commenting on the first of these lamented that:

“We really haven’t been able to keep up with walking the ground at our XXXXX, and so we just don’t know what kind of condition the network is in anymore.”

Six interviewees commented on how austerity had also impacted their human capital, especially the freezes in staff recruitment. Whilst there were not any formal redundancy programs, retirements were encouraged and vacancies, where they did emerge, were often left unfilled. This meant a reducing input of new talent into the agency and an ageing employee base, who whilst they enjoyed significant and increasing expertise were also as a body closer to retirement. This was, according to P11, a “*ticking timebomb waiting to go off in 2030*”. Four interviewees noted how this presents a future risk to agency where a critical mass of retirees was increasingly imminent, with the potential for lost expertise, institutional knowledge, stakeholder relationships, and regulatory competence across multiple fields of responsibility. This also meant the agency was less likely to recruit new staff to meet emerging challenge areas such as nature-based solutions to the climate crisis (P3). Though, considering that most of these emergent challenge areas were not covered by statutory regime and functions, and because austerity did not for them see an increase of new duties, this was less immediately problematic. Though others did perceive that the “*lack of fresh blood’*”(P7) made the aftermath of austerity, especially in light of the repatriation of regulatory duties through the UK’s leaving the EU particularly challenging. As P3 noted,

“Were living on borrowed time - without a real and sustained investment in new people there will come a point where XX (the agency) just ceases to be useful.”

Another issue concerned how austerity funding restrictions detriment existing staff development and training needs. Consequently, staff were less likely to be able to keep up with new techniques and skills and, as above, weren’t able to recruit the skills they could not train. Staff wages also failed to keep up with inflationary pressures let alone for professional development during this period, meaning participants reported that staff felt under-rewarded, under-recognized, and were working in an environment that did not share and support their personal-professional development goals.

Four interviewees discussed how the austerity decade slowly affected the culture of the agency with consequences on regulatory effectiveness. It was noted how the perception of reduced staff capabilities coupled with an impaired understanding of place-based governance conditions and dynamics increasingly bred a culture of reticence in regulatory enforcement. This cultural shift was influenced by other factors as well, including an increasing sense that they didn’t enjoy as much political support for adopting robust positions on regulatory enforcement, a growing awareness of the risks of litigation costs where robust enforcement decisions were challenged in court; and a growing awareness of their data deficiencies which compromised their decision making around regulatory enforcement. The corollary of these factors was a shifting agency culture towards non-interventionism and weak regulatory enforcement, or as commented by P10 “*We just don’t take the strong kind of positions on enforcement that we used to*”.

### Agency Resilience(s)

Interviewees offered perspectives on the organizational resilience in the wake of the austerity decade. Seven of these perspectives orientated around what might be described as broadly-defensive perspectives on agency resilience, and were various iterations of the comment by P9 of what they would be able to achieve “*once we’ve gotten back to where we were*”. Though there were two other comments (P7, P11) perceiving that there was not any chance of ever “getting back” to where the agency was in terms of capacities in 2010 (P11). Even with the seven comments about the challenges and routes back to pre-austerity levels of capacity the majority of these (*n* = 5) perceived that this return to capacity was unlikely within a decade (e.g., 2030) without significant funding and institutional support. Three participants argued that part of the problem in getting back to 2010 capacity was confounded by the multi-functionality of the agency. And that certain agency functions might recover faster than others—dependent upon how closely they were able to tie themselves to political agenda (P11). As noted by P4 rather despairingly “*regulation just isn’t sexy with this government*”; or as P10 commented “*things haven’t gone wrong enough yet for politicians to be impacted*”. This point was made in different ways by three interviewees—that the agency was only likely to see sufficient funding and support to get back to pre-austerity levels of capacity when the political consequences of regulatory bad governance were being felt. Certainly, interviewees felt it right and proper that the agency get back to pre-austerity capacities for meeting their regulatory functions as a matter of good governance, but that the politics of (re)funding were misaligned with normative ideas of good environmental regulatory governance.

Five interviewees thought that the austerity decade had opened up new opportunities for innovating and improving service delivery across all key functions. Three interviewees perceived it as an opportunity for innovation in leveraging new technologies and techniques. P5, for example, discussed the potential opportunity that came from relatively lower cost “*satellite altimetry data for landscape monitoring*”. Or P10 who discussed the potential opportunity of using drone technologies for meeting their statutory duties for collecting environmental data. A majority (*n* = 8) agreed that the perturbation caused by the austerity decade offered them the opportunity to reassess service delivery and meeting functions in lower-cost ways. P10 argued that this also opened up the potential to position the agency as leader in the delivery of new and emergent post-austerity/post-Brexit environmental meta-agenda. However, it was clear from four other comments that there was a significant skepticism in other interviewees about the agency using the perturbation as a springboard for delivering better and/or streamlined services, rooted in concerns about the impacts on their human capital.

Five interviewees commented on broadly anticipatory aspects on agency resilience. Three of them suggested that due to its proximity to government and policy-making processes they had a degree of foresight about impending shifts in policy. Such as P2 who suggested:

“As we’ve gotten closer to Defra were more inside the tent now, but that doesn’t always mean we can do anything about it”

This speaks to the fundamental challenge identified by interviewees, that as a public organization, no matter how ostensibly “*arms-length and independent*” (P7), their ability to make adjustments in anticipation of future perturbations was limited due to their strict functions and accountabilities; coupled to limitations placed upon them by under-funding and human capital limitations. Thus, whilst in theory the agency might structurally have the autonomy (under the aegis of agentification) to have anticipatory resilience, the strict and restrictive conditions that the operated under meant that they did not have significant opportunities to avoid potential perturbations. Two interviewees argued that this was not an absolute paucity of agency in anticipatorily avoiding perturbations. They had theoretical anticipatory resilience, but not in practice. As P9 noted there was limited room to maneuver in their advisory-expert functions: “*we started shaping what we were advising to line up with likely budgetary squeeze*”.

### Analysis

The analysis utilizes the Duchek’s conceptual framework as an analytical tool (Fig. 1) for parsing the findings as they relate to and answer the central research question.

### Bouncing Back

There was an initial need to ascertain if the austerity decade was considered a temporally bound organizational perturbation and in so doing, justify the conceptual framing in organizational resilience. The findings were emphatic—the participants found the austerity period of 2010–2020 a major perturbation to the four functions of the agency (congruent with Heald and Steel 2018; Kirsop-Taylor et al. 2020). A comment from P5, that “*austerity looked bad and felt worse*” bore out the real terms adjusted GiA of 67.8% seen in Fig. 3. The majority of the findings coalesced around a perception of austerity as a negative and prejudicial experience, with the agency adopting reactive reactions to it.

There was a significant (*n* = 7) participant perception that they *needed* to “bounce back” to meeting their regulatory functions, as these had been impaired by the funding reductions of austerity. This was a challenging conversation for many, as there was a tacit acceptance that regulatory functions should never have been allowed to lapse due to their strict (and in places statutory) natures, but that by mutual consent with central government there was an acceptance that these could be allowed to “*go soft*” (P7). That said, the overwhelming perception from interviewees was one of wanting, or aspiring, towards being defensively resilient (e.g., Horne and Orr 1998, Limnios et al. 2014) and bouncing back to, but not exceeding, meeting their regulatory functions. A common theme from interviewees was the need to minimize the time taken in bouncing back on regulatory functions—as the longer taken the worse the likely damage to the natural environment on their watch. But, critically, any sense of agency (will-to-action) in bouncing back on regulatory functions was not within their power as an organization—due to the nature of GiA and their limited tools to influence government fiscal agenda during the austerity decade (e.g., Kirsop-Taylor and Russell 2021). Indeed, many expressed a sense of frustration at the realization that bouncing back on regulatory functions was impractical in the hyper-politicized public funding environment (Ennser-Jedenastik 2016); until regulatory good governance through adequately funded and staffed regulatory activities was a politically salient subject (Shapiro and Morall III 2012). Bouncing back to 2010 levels of regulatory good governance was contingent on political agenda—and something they had limited influence on in agenda setting (Kirsop-Taylor and Russell 2021). That said, there were some who argued that there were already (in summer, 2020) signs of future political meta-agenda that the agency might use as a mechanism for bouncing back on regulatory functions, namely the increasing political salience of poor environmental water quality (similarly to Schaub 2019, but also Burns 2018) and for meeting the reinvigorated nature restoration agenda (UK Government 2020). The key challenge for the agency leadership (as noted by P9) was to use levers they had for framing these emergent politically salient agenda back towards regulatory good governance (Kirsop-Taylor and Russell 2021).

There was also an expressed hope that the agency might, at some point, bounce back to the level of substantive governance functions and positionality that they held in pre-austerity UK. P4 offered a clear articulation of the governance functions in the pre-austerity national environmental governance landscape—that they were both equal partners in partnership and delivery and also as an organizing and coordinating force. And that these two governance functions had both been damaged by the austerity decade. The expressed perspective of three interviewees was that bouncing back from their retreat from governance was primarily contingent upon the “general capacity” within the organization. That is, as a non-statutory, non-existential function for the agency, their governance functions had been allowed to wither during austerity; and that bouncing back was based upon having spare capacity within the organization *after* meeting their regulatory duties (which are mandated), their policy-making functions (which is a political priority), and their knowledge production functions (which goes hand-in-hand with governance). Their (pre-austerity) governance functions were considered as the “poor cousin” in the multi-functional aspects of the agency. That was not, as P5 argued to “*over-romanticize*“ the pre-austerity period, which certainly held its own challenges; but that scale of agency retrenchment from governance functions meant that the best that might be hoped for was a bounce back rather than bounce forwards in wider governance functions.

P4 argued that the challenge of “bouncing back” on governance was contingent upon (re)building the atrophied relationships with environmental voluntary sector organizations—the agency’s (former) trusted partners in national governance functions. They noted a perception of these relationships atrophying due to the agency’s increasing proximity to central government and re-positionality as an arbiter of scarce funding streams during austerity (Kirsop-Taylor et al. 2020). “Bouncing back” from this would require a significant, concerted, and multi-approach effort across the agency at rebuilding relationships and trust with voluntary sector governance partners. It would also require their re-adopting a clear leadership role in coordinating national environmental governance and partnerships. That said, P7 expressed a perception that, potentially, relationships with former governance partners in the environmental voluntary sector has been so badly damaged during austerity, and trust eroded so significantly, that there is no way to ever “bounce back” to where they were in 2010 as the main national locus for governance. And that, furthermore, with no real political focus on the value that the agency brought to the national environmental governance landscape anyway, that “bouncing back” without a funded, targeted, long-term organizational prioritization was unlikely.

### Bouncing Forwards

There was a perception (*n*-4) that Natural England would “bounce forwards” from the austerity decade (Lengnick-Hall et al. 2011, Limnios et al. 2014) in its policy functions; due in large part to its increasingly proximity to their ministerial department, Defra. Throughout the austerity decade the agency was drawn closer to the policy-making process (Boyer 1960, Verschuere 2009, Bach 2012, Bach et al. 2012), due to the need for post-Brexit replacement agri-environmental policy regime based on the expertise drawn from the agency’s experts (Burns 2018). Indeed, on the subject of Brexit which happened simultaneously with the later stages of the austerity decade, there was a perception that this offered an opportunity for the agency to “bounce forward” in their policy functions by evidencing their capacities and competencies supporting policymaking. Two interviewees perceived that they would emerge from the austerity decade stronger and closer to national policymaking and agri-environmental policy implementation. Though P4 described this increased competence in their policy functions as a “*double-edged sword*”: the more proximate to the locus of Defra policymaking they were orientated, the heightened risk to agency independence and autonomy, which was especially problematic if they were seeking to build bridges with the environmental voluntary sector for meeting governance functions. There was a delicate balance to be struck between acting as a trusted and expert national governance coordinator (the 2010 position) or staying closer to policy making with associated benefits to legitimacy and abilities to centrally champion policy issues (e.g., its advisory functions). P5 posed this as an un-resolved trade-off to be addressed; did the agency seek to “bounce forwards” from the austerity decade in its advisory and policy functions or instead seek to “bounce back” in its governance functions? Another interviewee offered a form of answer to this question in an oblique answer to a different question—that the organization, in fact, had limited agency in actually making a strategic decision in this trade-off; and that instead these kinds of outcomes are subject to more substantive political and global forces. In this way ‘bouncing forwards’ on the policy and advisory functions appeared a priority congruent with the increasingly politicized natures of agency governance (e.g., Ennser-Jedenastik 2016).

### Leaping Forwards

Interviewee perceived a lack of organizational anticipatory resilience towards the austerity decade. They argued this was partly structural, a function of the nature of public environmental organizations (Bach 2012, Elston 2012), and somewhat contingent upon environmental factors. Four interviewees noted how as a public agency with strict accountabilities, they are constrained in their abilities to adaptively and dynamically pivot their activities to suit the changing governance and public environment that they existed within. One interviewee suggested that the sheer weight and breadth of agency functions inhibited their adaptiveness and dynamism. Two others argued that their lack of anticipatory dynamism was a function of their limited political autonomy (Bach 2018). And P4 and P9 perceived that their lack of anticipatory resilience to “leap forwards” ahead of the austerity agenda was stymied by un-adaptive senior leadership decision-making (Stewart and O’Donnell 2007, Franken et al. 2020).

Two other interviewees argued that even through the agency enjoyed a small degree of foresight about impending perturbations, there wasn’t much they could do to act upon this as they had diminishing financial resources at the time. The data suggested that anticipatory resilience might still be considered as one approach-facet to organizational resilience, but it was one that was largely inappropriate in public environmental agency settings. This was due to the complex multi-functionality of such organizations coupled with uncertain political landscapes can make unfavorable conditions for adaptiveness even more problematic. Though, of course, this needs to be seen from the perspective of the different forms of public agency (Levi-Faur 2010, Jordana et al. 2018) replete with varying autonomy regimes.

### Analysis of Three-Part Framework of Organizational Resilience in Public Agencies

The data collected here have bearing on the public organizational resilience literature (Meyer 1982, Horne and Orr 1998, Boin and Eten 2013, Limnios et al. 2014). Duckek’s framework helpfully summarized the long-standing and rich organizational resilience literature into a three-part framework—structured around defensive (Limnios et al. 2014), offensive (Lengnick-Hall et al. 2011), and anticipatory (McManus et al. 2008, Somers 2009) resilience. In this paper, these were characterized as “bouncing back”, “bouncing forwards” and “leaping forwards” from the major perturbation of the austerity decade. The main finding suggests that large public environmental agencies are complex multi-functional organizations which defy easy characterization on this framework. Indeed, different functions of an agency will likely evidence different types and depths of resilience to perturbation. And resilience is complicated by the different organizational modalities that the function takes—for example, in an increasingly commercialized public space (see: Brown et al. 2000) particular environmental agency functions operating upon commercial-business lines will operate under different institutional logics compared to non-commercial strict regulatory functions. This implies that public agency resiliencies in the wake of perturbations are likely to disjointed, heterogenous, and un-coordinated. This un-coordination in overall agency resilience is further complicated by shifting environmental factors, in this case, the increasing politicization of agency governance. In short—for a public agency of the size and multi-functionality as Natural England there was limited opportunity for whole organizational “bouncing back” or “bouncing forwards” or “leaping forwards”. And whilst some agency functions were likely to “bounce forwards”, there remained a risk that some functions may never “bounce back” at all.

Consequently, whilst Duchek’s framework (2020) and the organizational literature it draws upon have clear theoretic value in describing private organizations and specific functions of larger public organizations, other conceptualizations from this literature (see: Tengblad and Oudhuis 2018) might offer a better fit for exploring resilience in large multi-functional environmental public agencies. That said, the three forms of resilience in the framework were shown to have descriptive value in characterizing how aspects of agency functions might bounce back, bounce forwards or even leap forwards from perturbations. Though the findings of this study suggest that any discussion or consideration of resilience in public agencies needs to try and take account of the different organizational logics, configurations, and multi-functionalities that determine and limit public organizations. Furthermore, this research highlights that resilience in environmental public agencies is intrinsically bound up in the political dimensions of public regulatory good governance (Ennser-Jedenastik 2016, Kirsop-Taylor and Russell 2021) and cannot be easily separated.

## Conclusion

Environmental public agencies are central actors in effective good governance of national natural environments, and they have leading-edge roles to play at the forefront of addressing some of the major environmental and climate challenges of the 21st century. Yet questions and discourse about the resilience of these agencies to political and economic perturbations have thus far been scant. This paper has addressed one aspect of this discourse—by highlighting the lasting impacts of the austerity decade of de-funding upon one of these agencies. The data collected addressed the central research question by evidencing that Natural England had been shown to be, broadly-speaking, un-resilient to the austerity decade. Though, critically, it also evidenced the unhelpfulness of crude single claims to resilience in an environmental public agency of this size and with the forms of operational complexity it operates within. Instead, the different functions that the agency operates under and has responsibilities for experience their own unique forms of resilience based upon issues of political priorities, organizational capacities, and environmental conditions. In the wake of major perturbations, such as the austerity decade, public organizational resilience should instead be seen as a complex and dynamic mosaic of heterogenous forms of resilience—with some “bouncing back”, “bouncing forwards”, and “leaping forwards”. The results of this research with the senior management of Natural England suggest its own mosaic of resilience—whilst it will likely use the austerity decade to “leap forwards” in better meeting and delivering its policy and advisory functions; it might take far longer to “bounce back” to its level of capacity in meeting its regulatory functions; but worse it is likely to take significant concerted and strategic efforts to “bounce back” to its 2010 capacity and positionality in national governance (convening and partnering). Though this is perhaps itself a disingenuous argument: as the governance landscape evolved so too must the agency and efforts to bounce back to 2010 capacity levels are perhaps purely nostalgic. And that it is far better to engage in a continual process of change and renewal congruent to institutional landscape that it operates within.
